# Dendritic cell-based vaccine prepared with recombinant *Lactococcus lactis* enhances antigen cross-presentation and antitumor efficacy through ROS production

**DOI:** 10.3389/fimmu.2023.1208349

**Published:** 2023-08-30

**Authors:** Tingting Zhang, Xianxian Wei, Yijie Li, Shuai Huang, Yulin Wu, Shanshan Cai, Adila Aipire, Jinyao Li

**Affiliations:** Xinjiang Key Laboratory of Biological Resources and Genetic Engineering, College of Life Science and Technology, Xinjiang University, Urumqi, China

**Keywords:** recombinant *Lactococcus lactis*, dendritic cell-based vaccine, reactive oxygen species, cross-presentation, antitumor efficacy

## Abstract

**Introduction:**

*Lactococcus lactis* (*L.L*) is safe and can be used as vehicle. In this study, the immunoregulatory effect of *L.L* on dendritic cell (DC) activation and mechanism were investigated. The immune responses and antigen cross-presentation mechanism of DC-based vaccine prepared with OVA recombinant *L.L* were explored.

**Methods:**

Confocal microscopy and flow cytometry were used to analyze the mechanism of *L.L* promoting DC maturation, phagosome membrane rupture and antigen presentation. The antitumor effect of DC vaccine prepared with *L.L-OVA* was assessed in the B16-OVA tumor mouse model.

**Results:**

*L.L* significantly promoted DC maturation, which was partially dependent on TLR2 and downstream MAPK and NF-κB signaling pathways. *L.L* was internalized into DCs by endocytosis and did not co-localized with lysosome. OVA recombinant *L.L* enhanced antigen cross-presentation of DCs through the phagosome-to-cytosol pathway in a reactive oxygen species (ROS)- and proteasome-dependent manner. In mouse experiments, *L.L* increased the migration of DCs to draining lymph node and DC vaccine prepared with OVA recombinant *L.L* induced strong antigen-specific Th1 and cytotoxic T lymphocyte responses, which significantly inhibited B16-OVA tumor growth.

**Conclusion:**

This study demonstrated that recombinant *L.L* as an antigen delivery system prepared DC vaccine can enhance the antigen cross-presentation and antitumor efficacy.

## 1 Introduction

Dendritic cells (DCs) with high degree of heterogeneity are differentiated from myeloid and lymphoid stem cells. Distinct DC populations have different phenotypical and functional properties (1). Conventional DCs (cDCs) can migrate from tumor tissues to draining lymph nodes (LNs) and activate naïve T cells to differentiate into antigen-specific effector or memory T cells, which play the role of immune surveillance and clearance of tumor cells (2, 3). Among them, cDC1s can effectively activate tumor-specific CD8^+^ cytotoxic T lymphocytes (CTL) and cDC2s can induce Th17 responses, which inhibit tumor growth (4). The first clinical trial of plasmacytoid DC (pDC) vaccine had shown that several melanoma patients generated antigen-specific CD4^+^ and CD8^+^ T cell responses (5).

DCs are professional antigen presenting cells, which mainly present exogenous antigens through MHC II pathway to activate CD4^+^ T cells. However, some exogenous antigens can also be displayed by MHC I pathway to activate CD8^+^ T cells and differentiate into CTL, known as antigen cross-presentation (6, 7), which effectively clear bacterial, viral and tumor antigens (8). According to the location of antigen processing, antigen cross-presentation can be divided into transporter associated with antigen processing (TAP) and proteasome-dependent pathway, TAP and proteasome-independent pathway, and TAP-independent proteasome processing pathway (9–11). TAP and proteasome-dependent pathway is known as the phagosome-to-cytosol pathway (6). After fusion of endosome contained the internalized exogenous antigen with lysosome, part of the exogenous antigen enters the cytoplasm through the endoplasmic reticulum (ER)-associated degradation (ERAD) mechanism (12, 13) and is degraded by the proteasome (14), then these peptides are transported to ER or phagosomes by TAP and bound to MHC I (15). The MHC I-peptide complex is transported to the cell surface to activate CD8^+^ T cells (16). TAP and proteasome-independent pathway is known as the vacuolar pathway (17). The exogenous antigen is degraded by cathepsin S in the phagosomes (18), and the generated antigenic peptide binds to MHC I in the phagosomes (19). In TAP-independent proteasome processing pathway, proteasomes in phagosomes generated antigenic peptides and bound to MHC I in phagosomes.

To promote the escape of exogenous antigens from phagosomes to cytoplasm is one of the mechanisms to enhance antigen cross-presentation through TAP and proteasome-dependent pathways (20, 21). Cell-penetrating peptide can promote endosomal escape and antigen cross-presentation (22). *Listeria* monocytogenes secretes listeriolysin O, which destroys the phagosome membrane during acidification and promotes the escape of antigens and DNA from the phagosomes into the cytoplasm (23). Ovalbumin (OVA) coupled with osmotin can escape from the phagosomes into the cytoplasm or directly transfer into the cytoplasm (24). With maturation of DCs, reactive oxygen species (ROS) generated by NADPH oxidase 2 (NOX2) can promote phagosome membrane rupture and exogenous antigen escape to increase antigen cross-presentation (13, 25). *Lactococcus lactis* (*L.L*) is an FDA approved probiotic, which can be used as vehicle to deliver antigens in various ways (26). *L.L* can induce DC maturation, promote Th1 cell differentiation and increase the expression of IL-12, IFN-γ and TNF-α through TLR2, TLR3 and TLR9 pathways (27–30). Therefore, *L.L* as antigen delivery vehicle can be used to prepare DC-based vaccine and enhance antigen cross-presentation due to its adjuvant effect.

Here, we investigated the adjuvant effect and mechanism of *L.L*, and antigen cross-presentation and immune responses of GM-DC-based vaccine prepared with OVA recombinant *L.L*. Its anticancer effect was evaluated in a B16-OVA tumor mouse model. We demonstrated that *L.L* enhanced GM-DC maturation through TLR2 and downstream MAPK and NF-κB signaling pathways and produced high levels of ROS, which promoted phagosome membrane rupture and antigen escape into the cytoplasm to increase antigen cross-presentation through the phagosome-to-cytosol pathway. The GM-DC-based vaccine induced strong Th1 and CTL responses and significantly inhibited tumor growth. Our study provided a potential vaccine strategy for cancer immunotherapy.

## 2 Materials and methods

### 2.1 Animals

6 to 8 weeks of C57BL/6 male mice were bought from Animal Laboratory Center, Xinjiang Medical University (Urumqi, Xinjiang, China) and housed in an animal facility of Xinjiang University. The experimental protocols were approved by the Committee on the Ethics of Animal Experiments of Xinjiang Key Laboratory of Biological Resources and Genetic Engineering (BRGE-AE001).

### 2.2 Plasmids, bacterial strains and growth conditions

Plasmids including pMG36e-*RFP-EGFP* (358), pMG36e-*EGFP-mcherry*, pMG36e-*penp-OVA*, pMG36e-*CMV-penp-OVA* and pNZ8149-*penp-OVA* were constructed and kept by Xinjiang Key Laboratory of Biological Resources and Genetic Engineering. pMG36e-*RFP-EGFP* (358) encodes a green fluorescent protein (EGFP) under the control of a eukaryotic promoter CMV and a red fluorescent protein (RFP) under the control of a prokaryotic promoter penp. pMG36e-*EGFP-mcherry* encodes EGFP-mcherry under the control of a prokaryotic promoter penp. pMG36e-*penp-OVA* encodes OVA protein under the control of penp. pMG36e-*CMV-penp-OVA* encodes OVA protein under the control of CMV and penp. pNZ8149-*penp-OVA* encodes OVA protein under the control of penp. The *L.L* was cryopreserved in this laboratory. *L.L* and recombinant *L.L* carrying pMG36e were grown statically at 30°C in M17 broth supplemented with 10% glucose (GM17), recombinant *L.L* of pMG36e was grown in GM17 with 1 mg/ml erythromycin (Solarbio, China). Recombinant *L.L* carrying pNZ8149 was grown statically at 30°C in M17 broth supplemented with 10% lactin, recombinant *L.L* of pNZ8149 were grown in M17 with lactin and 50 μg/ml ampicillin (Solarbio, China).

### 2.3 Genetic transformation

*L.L* at logarithmic (log) phase was collected and resuspended in 1 ml lithium acetate solution (100 mM LiAc; 10 mM DL-Dithiothreitol; 0.6 M sucrose; 10 mM Tris-HCl, pH 7.5) (Shenggong, Shanghai, China). After incubation for 30 min at room temperature, cells were washed three times with sterile deionized water and resuspended in 2 ml of 0.3 M sucrose with 10% glycerol. Cells can be used for electroporation or stored at -80°C for future use.

1 μg of plasmid was mixed with 80 μl cells in a 2 mm electroporation cuvette (BIO-RAD, USA). The voltage of BTX electro cell manipulator was set at 400 V, the time was 4 ms and post fusion AC seconds were 1 (Harvard apparatus, USA). After electroporation, 1ml recovery medium (900 μl M17, 100 μl 5% glucose or 5% lactin, 10 μl 20 mM MgCl_2_ and 2 mM CaCl_2_) (Shenggong, Shanghai, China) was added and the cells were kept on the ice for 5 min and incubated at 30°C for 2 h. After centrifugation at 4000 rpm for 5 min, part of the supernatant was discarded, 200 μl cells were plated on Elliker-medium (EM) with 1mg/ml erythromycin or 50 μg/ml ampicillin and cultured for 24-48 h at 30°C.

### 2.4 Transfection of 293T cells

The 293T cells were cultured in 6 well plates and transfected when the cell density was 60%-70%. Before the transfection, the culture medium was replaced with fresh one. The transfection system was included A (2 μg plasmid, 125 μl Opti-MEM) and B [3.75 μl Lipo3000, 4 μl P3000, 125 μl Opti-MEM (Thermo Fisher, USA)]. The A and B tubes were evenly mixed and kept at room temperature for 15 min. The mixture was slowly added to 6 well plates and mixed. The culture medium was replaced after 8 h. After 36 h, cells were collected and total protein was isolated by the RIPA Lysis Buffer (Beijing ComWin Biotech Co., Ltd). The expression of related proteins was detected by Western blot. The expression of EGFP proteins was also observed by inverted fluorescence microscope (Nikon, Japan).

### 2.5 Western blot

For analyzing the activation of MAPK and NF-κB signaling pathways, GM-DCs were treated with 10^2^ *L.L* and 10^3^ *L.L* for 30* min* or 120 min and proteins were extracted by nucleoprotein and cytoplasmic protein extraction kit (Biorebo Biotech, China). 40 ng/mL LPS (Sigma-Aldrich, USA) was used as the positive control.

For detection of EGFP and OVA expression, the recombinant *L.L* with pNZ8149*-penp-OVA* (*L.L-OVA)* was cultured at 30°C to OD_595nm_ of 0.2-0.6. Then 10 ng/ml nisin was added and cultured for 0 h, 9 h, 12 h and 24 h at 30°C. The recombinant *L.L* with pMG36e is auto-inducible. The recombinant *L.L* was harvested by centrifugation (12000 rpm, 10 min), and the proteins were obtained by liquid nitrogen grinding. The expression of related proteins was detected by Western blot, including EGFP (TransGen Biotech, China) and OVA (Elabscience, China). After incubation with HRP-conjugated goat anti-mouse IgG, or HRP-conjugated goat anti-rabbit IgG (TransGen Biotech, China), the target proteins were detected using EasySee Western blot kit (TransGen Biotech, China).

### 2.6 *DC treatment*, *flow cytometry* and cytokine detection

Granulocyte/macrophage colony-stimulating factor (GM-CSF) derived DCs (GM-DCs) were induced from bone marrow cells of C57BL/6 mice referring to our previous description (31). Briefly, bone marrow cells were isolated from femurs and tibias of C57BL/6 mice and cultured in RPMI-1640 containing 10% fetal bovine serum, 1% penicillin-streptomycin, 2 mM L-glutamine, 50 μM β-mercaptoethanol and 20 ng/mL GM-CSF (PeproTech) at 37°C in 5% CO_2_ incubator. Medium was changed every other day. On day 7, cells were collected and counted by blood cell count plate counting. CD11c^+^CD86^+^ were used to identify GM-DCs following differentiation. GM-DC populations were analyzed by flow cytometry after staining with anti-CD11c-FITC, anti-CD11b-PerCP/Cyanine5.5, anti-CD45-PE, anti-CD103-APC (Elabscience, China) and anti-CD24-v450 (Thermo Fisher, USA) according to previous studies (4, 32). 5 × 10^5^ GM-DCs were treated with different concentrations (10, 10^2^, 10^3^) of *L.L* for 12 h, 24 h or 48 h. 40 ng/mL LPS (Sigma-Aldrich, USA) was used as the positive control. Cells were collected and stained with fluorescence-labeled antibodies including anti-CD40-FITC, anti-CD86-APC, anti-CD80-APC, anti-MHC I-FITC, anti-MHC II-APC (Elabscience, China) or anti-25-D1.16-APC (Thermo Fisher, USA). Apoptosis of GM-DCs was analyzed by AnnexinV-FITC/propidium iodide (PI) apoptosis detection kit according to the manufacturer’s instruction (YEASEN, China). ROS production was measured by the reactive oxygen species assay kit according to the manufacturer’s instruction (Biyuntian, China), and cells were stained with DCFH-DA. For detection of GM-DC endocytosis, GM-DCs were treated with 10^3^ fluorescent protein recombinant *L.L* or CFSE-stained *L.L*. Samples were collected by FACSCalibur (BD Biosciences, USA) and analyzed by FlowJo (Tree Star, Inc., Ashland, OR).

To investigate the roles of TLR2/4 in GM-DC maturation, cells were pretreated with 100 ng/mL TLR2 blocking antibody (mAb-TLR2) or 1 μM TAK-242 (TLR4 inhibitor) for 1 h, and washed with PBS, then treated with different doses (10, 10^2^, 10^3^) of *L.L* for 24 h. For NOX2 inhibitor, ROS scavenger and endocytic inhibitor treatment, GM-DCs were pretreated with 5 μM Diphenyleneiodonium chloride (DPI) (Medchem-express, USA), 10 mM N-Acetylcysteine (NAC) (Sigma-Aldrich, USA) and different doses (0.1, 0.3, 0.5 and 0.7 μg/ml) of cytochalasin D for 12 h (Yuanye, China), then treated by 10^3^ *L.L* or recombinant *L.L* for 6 h or 24 h. For proteasome inhibitor treatment, GM-DCs were pretreated with 10 μg/ml MG132 for 30 min and co-treated with 10^3^ *L.L-OVA* for 24 h, then another 10 μg/ml MG132 was added after 12 h.

To further confirm the roles of MAPK, JNK and ERK in *L.L*-induced GM-DC maturation, GM-DCs were pretreated with inhibitors of p38 MAPK (SB202190), ERK (U0126) and JNK (SP600125) for 2 h, 1 h and 45 min, respectively, and then treated with 10^3^ *L.L* for 24 h.

After treatment, the supernatant was collected to detect cytokine secretion by enzyme-linked immunosorbent assay (ELISA) using an ELISA kit according to the manufacturer’s instruction (Boster, China) and nitric oxide (NO) by the NO colorimetric assay kit according to the manufacturer’s instruction (Biyuntian, China).

### 2.7 Confocal laser scanning microscopy

To evaluate the intracellular localization of the CFSE-stained *L.L* (*L.L-*CFSE) and EGFP-mcherry, GM-DCs were treated with 10^3^ *L.L-*CFSE and recombinant *L.L* with pMG36e-*EGFP-mcherry* (*LEGFP-mcherry*). After treatment for 2 h, 4 h or 6 h, cells were collected and fixed with 4% paraformaldehyde at room temperature for 10 min, then washed with PBS for 3 times. After treatment with 0.1% trionx-100, EEA1 and LAMP1 (diluted with PBS at 1:100; Affinity, USA) were added and incubated at 4°C overnight. After washing with PBS for 3 times, goat anti-rabbit IgG (diluted with PBS at 1:300) was added and incubated at 37°C for 2 h. Then DAPI was added and kept at room temperature for 10 min (Biyuntian, China) after washing with PBS. Finally, samples were imaged using laser confocal microscope (Nikon, Japan).

### 2.8 GM-DC migration experiment

GM-DC treated with (10, 10^2^, 10^3^) *L.L*, 40 ng/mL LPS was used as a positive control. 2×10^5^ GM-DC (cells resuspended in 0.5 mL complete RPMI-1640 medium) was added to the upper chamber of transwell, and 0.5 mL complete RPMI-1640 medium containing 100 ng/mL CCR19 was added to the lower chamber of transwell. After 3 h, cells in the subchamber of transwell were collected and counted.

GM-DC migration *in vivo* was performed as previously described (33). Briefly, GM-DC treated with 10^3^ *L.L*, 10^3^ recombinant *L.L*, or 40 ng/mL LPS were stained with 10 μM CFSE and injected in footpad of mice. After 24 h, popliteal LNs were isolated to analyze CFSE^+^ GM-DCs by flow cytometry.

### 2.9 Preparation of GM-DC-based vaccine

GM-DC-based vaccine was prepared according to our previous study (34). Briefly, GM-DCs were treated with 10^3^ *L.L*, 10^3^ recombinant *L.L* or 40 ng/mL LPS for 24 h and then the LPS groups were incubated with 10 μg/ml OVA_257-264_ and OVA_323-339_ peptides, or 10 μg/ml OVA protein for 2 h, respectively. GM-DCs treated with *L.L* were used as negative control.

### 2.10 Evaluation of immune responses and antitumor effect of GM-DC-based vaccine

Naïve C57BL/6 mice (5 mice/group) were intradermally injected with GM-DC-based vaccines (1 × 10^6^ GM-DCs in 50 μl PBS) twice at 2 weeks intervals, including LPS treated GM-DCs pulsed with OVA protein (LPS+OVA), LPS treated GM-DCs pulsed with OVA peptides (LPS+OVApep), *L.L* treated GM-DCs (*L.L*), *L.L-OVA* treated GM-DCs (*L.L-OVA*), MG132 pretreated GM-DCs treated with *L.L-OVA* (*L.L-OVA*+MG132) and DPI pretreated GM-DCs treated with *L.L-OVA* (*L.L-OVA*+DPI). 7 days after the second injection, mice were sacrificed to isolate spleens and inguinal LNs to detect immune responses. Splenocytes were stained with fluorescence-labeled antibodies against CD3, CD19, CD49b, CD11c, CD86, CD11b, Ly6C, Ly6G, CD4, CD25 and Foxp3 (BD Biosciences), and lymphocytes were stained with fluorescence-labeled antibodies against CD4, CD8, IFN-γ and Granzyme B (BD Biosciences).

For evaluation of antitumor effect of GM-DC-based vaccine, 5 × 10^5^ B16-OVA cells were subcutaneously injected into the right flank of C57BL/6 mice. After 6 d, tumor mice were randomly divided into 6 groups (6 mice/group) and treated with GM-DC-based vaccines including LPS+OVA, LPS+OVApep, *L.L*, *L.L-OVA*, *L.L-OVA*+MG132 and *L.L-OVA*+DPI. GM-DCs were intradermally injected into mice twice at 1 week interval. Tumors were measured every other day using callipers and calculated using the following formula: tumor volume (mm^3^) = (length × width^2^)/2. On day 24, mice were sacrificed, and organs and tumors were isolated and weighted. Inguinal LNs were isolated to detect immune responses according to the above description.

### 2.11 Statistical analysis

Data were reported as mean ± standard error of the mean (SEM). One-way analysis of variance (ANOVA) or a paired *t*-test was used to examine the statistical difference. Data were analyzed with Prism5 GraphPad software (USA). *p* < 0.05 was considered to be statistically significant.

## 3 Results

### 3.1 *L.L* promotes GM-DC maturation through TLR2 and downstream MAPK and NF-κB signaling pathways

According to the growth curve (**Supplemental Figure 1A**), colony-forming unit (CFU) calculation of *L.L* was carried out when OD_595nm_ was 0.5 at the log phase. The number of viable bacteria per milliliter of the bacterial solution was 10^10^ by counting the plate clone numbers and the CFU is 10^10^ CFU/ml. Firstly, the safe doses of *L.L* were determined based on the viability of GM-DCs. After treatment with different doses (10, 10^2^, 10^3^) of *L.L* for 24 h, GM-DC viability was not affected according to the frequencies of apoptotic and necrotic GM-DCs (**Supplemental Figure 1A**), suggesting that the selected doses is safe. Secondly, the phagocytosis ability of GM-DCs for *L.L* was detected. Compared with *L.L*-treated GM-DCs, the fluorescence intensities of *L.L-*CFSE-treated GM-DCs were significantly increased at 3 h, 6 h and 9 h (**Supplemental Figure 1B**). The similar results were observed by laser confocal microscope (**Supplemental Figure 1C**). Thirdly, the effect of *L.L* on GM-DC maturation was measured. After treatment for 24 h, *L.L* significantly increased the expression of CD40, CD86, CD80, MHC I, MHC II and CCR7, and the secretion of TNF-α, IL-1β, IL-12p40 and IL-6, in a dose-dependent manner. The results of transwell experiment *in vitro* showed that *L.L* can significantly promote GM-DC migration (**Supplemental Figure 2A**). Consistently, 10^3^ *L.L* significantly enhanced GM-DC migration *in vivo*, characterized by the increased frequencies of CD11c^+^CFSE^+^ cells in draining LN (**Supplemental Figure 2B**). Moreover, high doses of *L.L* significantly upregulated NO production (**Supplemental Figure 2C**). The results demonstrated that *L.L* promoted GM-DC maturation and activation.

**Figure 1:**
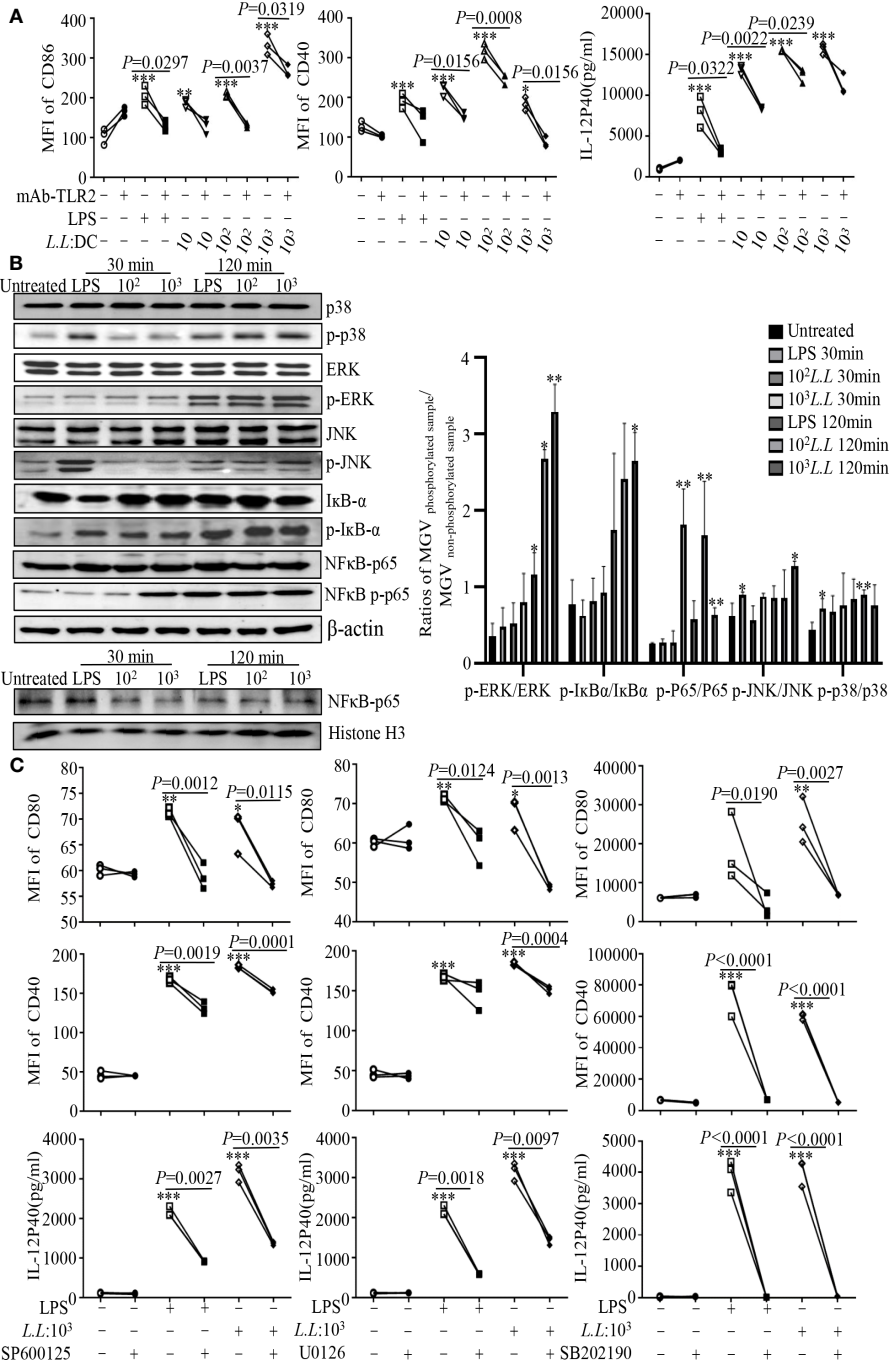
*L.L* enhanced GM-DC maturation by TLR2 signaling pathway. **(A)** GM-DCs were pretreated with mAb-TLR2 (blocking antibody) for 1 h, and then treated with LPS and *L.L* for 24 h. The expression of CD40 and CD86 were tested by flow cytometry. The IL-12p40 secretion was measured by ELISA. **(B)** GM-DCs were treated with *L.L* for 30 min and 120 min, then proteins of GM-DCs were isolated to detect their expression and phosphorylation by Western blot. **(C)** GM-DCs were pretreated with JNK inhibitor SP600125, ERK inhibitor U0126 and p38 MAPK inhibitor SB202190 for 45 min, 1h and 2 h, and then treated with LPS and *L.L* for 24 h. The expression of CD40, CD80 and IL-12p40 was measured. * *p* < 0.05; ** *p* < 0.01; *** *p* < 0.001 compared to untreated group.

To investigate the mechanism of GM-DC maturation, cells were pretreated with or without TLR2 blocking antibody (mAb-TLR2) for 1 h, and then treated with different doses (10, 10^2^, 10^3^) of *L.L* for 24 h. The expression of CD40, CD86 and IL-12p40 in GM-DCs induced by *L.L* was significantly reduced by mAb-TLR2 pretreatment (**Figure 1A**), indicating that *L.L* promoted GM-DC maturation partially through TLR2 signaling pathway. The expression and activation of key molecules in TLR2 downstream pathways were further analyzed by Western blot. *L.L* treatment significantly enhanced the phosphorylation of p38, ERK, JNK and IKB-α at 120 min and the phosphorylation of NF-κBp65 at 30 min and 120 min (**Figure 1B**). To further confirm the roles of p38, JNK and ERK in *L.L*-induced GM-DC maturation, GM-DCs were pretreated with inhibitors of p38 MAPK (SB202190), ERK (U0126) and JNK (SP600125) for 2 h, 1 h and 45 min, respectively, and then treated with *L.L* for 24 h. SP600125, U0126 and SB202190 pretreatment significantly inhibited the expression of CD40, CD80 and IL-12p40 (**Figure 1C**). TLR4 inhibitor (TAK-242) pretreatment did not suppress the expression of CD40, CD86 and IL-12p40 in GM-DCs induced by *L.L* but significantly inhibited their expression induced by LPS (**Supplemental Figure 3**). These results suggested that *L.L* promoted GM-DC maturation through TLR2 and downstream MAPK and NF-κB signaling pathways.

### 3.2 Construction and identification of recombinant vectors expressed fluorescent proteins and OVA

In order to study the mechanism of antigen processing and presentation delivered by *L.L* in GM-DCs, recombinant vectors expressing fluorescent proteins including pMG36e-*RFP-EGFP* (358) and pMG36e-*EGFP-mcherry* were constructed and protein expression was identified by Western blot and confocal laser scanning microscopy. For identification of EGFP expression of pMG36e-*RFP/eGFP* (358) under the control of CMV, the plasmid was encapsulated by liposome and transfected into 293T cells, then the protein was extracted and identified by Western blot. The expected band of EGFP with 29 KDa was obtained. The EGFP fluorescence was further observed in 293T cells by confocal laser scanning microscopy (**Supplemental Figure 4A**). pMG36e-*RFP/eGFP* (358) and pMG36e-*EGFP-mcherry* were transferred into *L.L* and named as *L358* and *LEGFP-mcherry*, which were confirmed by PCR (**Supplemental Figures 4A, B**). The expression of *EGFP-mcherry* in recombinant *L.L* was identified by Western blot. The expected band of EGFP-mcherry with 55 KDa was obtained (**Supplemental Figure 4B**). After co-culture of *LEGFP-mcherry* and GM-DCs, green fluorescence was observed in GM-DCs by confocal laser scanning microscopy (**Supplemental Figure 4B**), suggesting that *LEGFP-mcherry* was endocytosed into GM-DCs.

Recombinant vectors expressed OVA including pMG36e-*penp-OVA*, pMG36e-*CMV-penp-OVA* and pNZ8149-*penp-OVA* were constructed and transferred into *L.L*, which were named as *LpMG36e-penp-OVA*, *LpMG36e-CMV-penp-OVA* and *L.L-OVA*, respectively, and confirmed by PCR (**Supplemental Figures 4C–E**). OVA expression of *LpMG36e-penp-OVA* was identified by Western blot and the correct band was observed. For identification of OVA expression by pMG36e*-CMV-penp-OVA*, the plasmid was encapsulated by liposome and transfected into 293T cells, and then OVA protein band was obtained by Western blot. *L.L-OVA* was induced by nisin for 9 h, 12 h and 24 h, respectively, and the protein expression was detected by Western blot. The correct bands were observed (**Supplemental Figures 4C–E**).

### 3.3 Intracellular localization of EGFP proteins delivered by L.L

**Figure 2:**
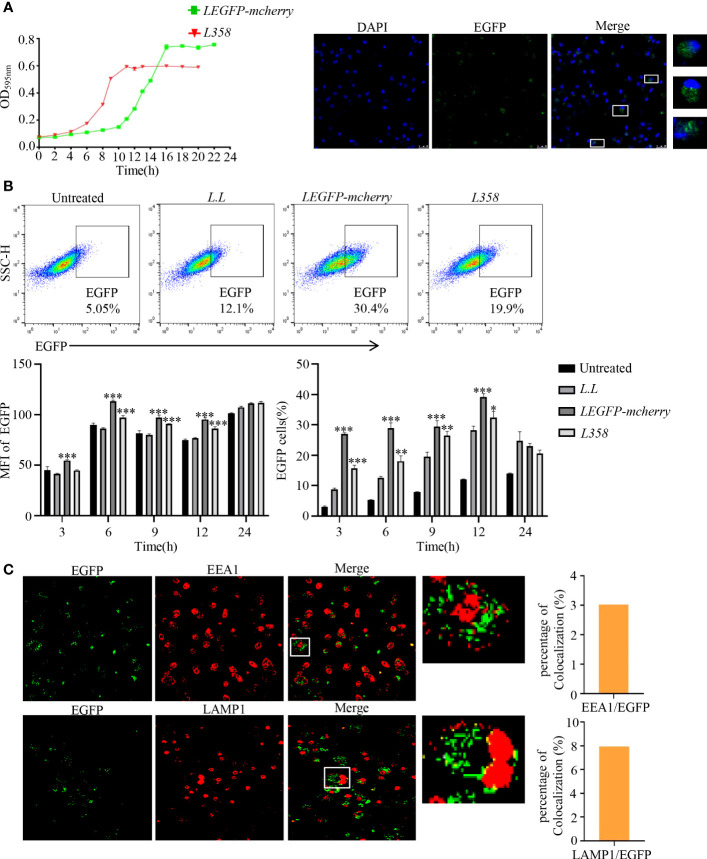
The expression of EGFP and its subcellular localization in GM-DCs. **(A)** Determination of growth curve of recombinant *L.L*. **(B)** Detection of EGFP expression in GM-DCs by flow cytometry and confocal laser scanning microscopy after co-cultured with recombinant *L.L* for 3 h, 6 h, 9 h, 12 h and 24 h. The time of the EGFP signal gating strategy is 6 h. **(C)** The co-localization of EGFP with EEA1 and LAMP1 was detected by confocal laser scanning microscopy. The higher magnification of areas in white boxes and quantification of colocalization were shown in right panel. * *p* < 0.05, ** *p* < 0.01; *** *p* < 0.001 compared to *L.L* group.

According to the growth curve (**Figure 2A**), *LEGFP-mcherry* and *L358* entered the log phase around 10 h and 4 h, respectively, and reached the plateau around 16 h and 10 h, respectively. *LEGFP-mcherry* and *L358* at log phase were used to treat GM-DCs for 3 h, 6 h, 9 h, 12 h and 24 h, and the fluorescence intensities of EGFP were detected by flow cytometry. Compared with untreated control, the fluorescence intensities of EGFP were significantly increased from 3 h to 12 h in GM-DCs treated with *LEGFP-mcherry* and *L358* (**Figure 2B**), suggesting that antigen not only was delivered into GM-DCs by *L.L* but also sustained at least for 12 h. EGFP fluorescence observed in GM-DCs treated with *L358* demonstrated that pMG36e-*RFP-EGFP* (358) was released into the cytoplasm due to EGFP under the control of CMV. We further observed that EGFP proteins were dispersed in GM-DCs treated with *LEGFP-mcherry* for 6 h (**Figure 2B**), and did not co-localize with EEA1 and LAMP1 by laser confocal microscope (**Figure 2C**). These results suggested that *L.L* promoted the rupture of the phagosome membrane to release antigen into cytoplasm.

### 3.4 Mechanism of GM-DC phagosome membrane rupture induced by L.L

**Figure 3:**
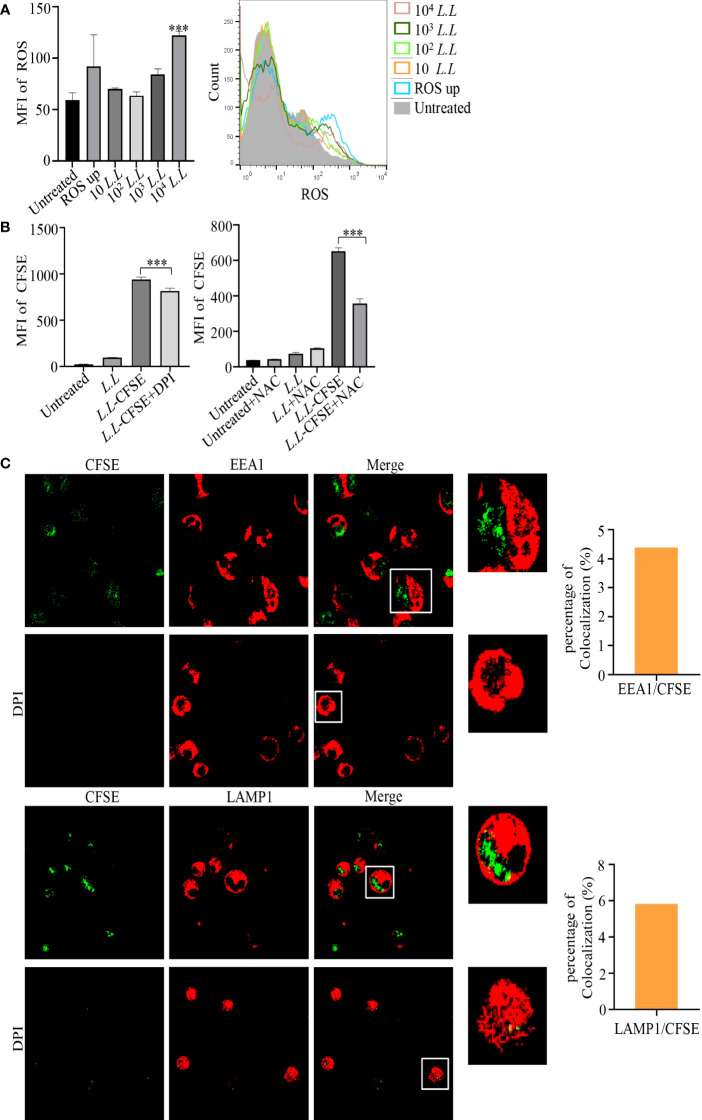
ROS production and amount of *L.L*-CFSE in GM-DCs. **(A)** The generation of ROS in GM-DCs was detected by flow cytometry after co-cultured with different concentrations of *L.L* for 6 h. **(B)** The fluorescence intensity of *L.L*-CFSE was detected by flow cytometry after the addition of DPI and NAC for 12 h. DPI inhibits ROS production in phagosomes. NAC inhibits all ROS production in cells. **(C)** CFSE subcellular localization and fluorescence intensity in GM-DCs were analyzed by confocal laser scanning microscopy after co-cultured with *L.L-CFSE* for 6 h in the presence or absence of DPI. The higher magnification of areas in white boxes and quantification of colocalization were shown in right panel. *** *p* < 0.001 compared to untreated group, or between indicated groups.

High levels of ROS can mediate phagosome membrane rupture and release antigens into the cytoplasm to enhance the antigen cross-presentation of GM-DC via the phagosome-to-cytosol pathway (25). Therefore, ROS production was measured in GM-DCs by flow cytometry after treatment with different doses of *L.L* for 6 h. As shown in **Figure 3A**, ROS production was significantly increased in a concentration-dependent manner. To explore the role of ROS production in antigen processing, NOX inhibitor, DPI, was used to suppress ROS production in phagosomes. We found that 5 μM DPI significantly reduced the fluorescence intensity of CFSE in GM-DCs treated with *L.L*-CFSE (**Figure 3B**). NAC (10 mM), a broad spectrum ROS inhibitor, also significantly decreased the fluorescence intensity of CFSE in GM-DCs treated with *L.L-*CFSE (**Figure 3B**).

**Figure 4:**
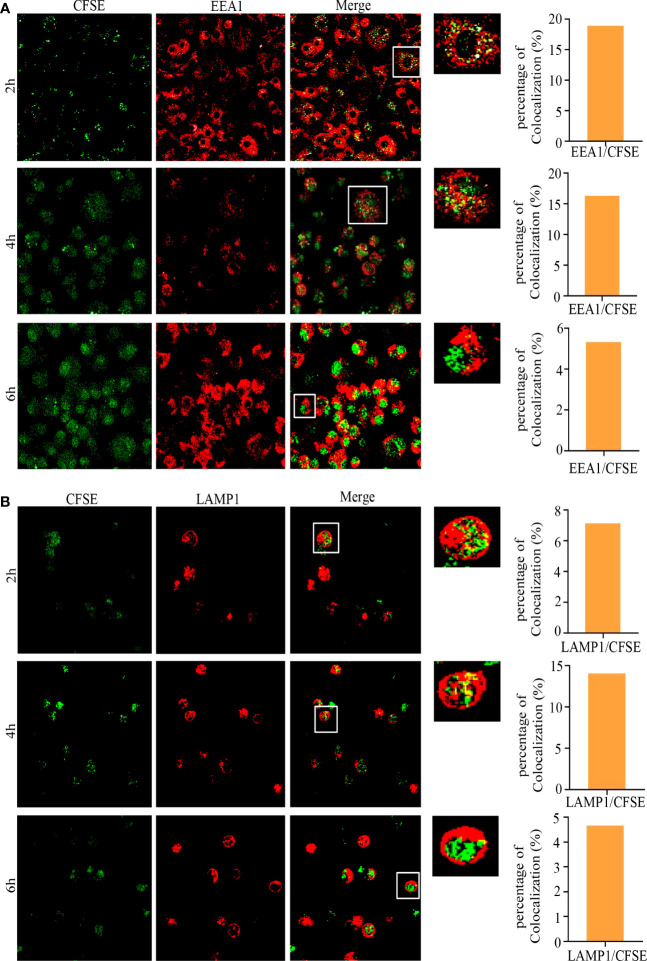
Subcellular localization of *L.L*-CFSE in GM-DCs. **(A)** Co-localization of *L.L*-CFSE and EEA1 after co-cultured with GM-DC for 2 h, 4 h and 6 h. **(B)** Co-localization of *L.L*-CFSE and LAMP1 after co-cultured with GM-DC for 2 h, 4 h and 6 h. The higher magnification of areas in white boxes and quantification of colocalization were shown in right panel.

We further analyzed the subcellular localization of *L.L*-CFSE with EEA1 and LAMP1 in GM-DCs treated with or without DPI by confocal laser microscope. *L.L*-CFSE was observed without DPI treatment and did not co-localize with EEA1 and LAMP1. This is consistent with the results in **Figure 3C**. However, no *L.L*-CFSE was observed after DPI treatment, indicating that most of the *L.L*-CFSE was degraded in GM-DCs (**Figure 3C**). The dynamic subcellular localization of *L.L*-CFSE was further investigated in GM-DCs. Cells were treated with *L.L*-CFSE for 2 h, 4 h and 6 h and stained with EEA1 and LAMP1, then observed by laser confocal microscope. As shown in **Figure 4A**, *L.L*-CFSE co-localized with EEA1 at 2 h, some *L.L*-CFSE co-localized with EEA1 and others leaked into the cytoplasm at 4 h, and almost *L.L*-CFSE did not co-localize with EEA1 and leaked into the cytoplasm at 6 h. *L.L*-CFSE did not co-localize with LAMP1 at 2 h, but some *L.L*-CFSE co-localized with LAMP1 and others leaked into the cytoplasm at 4 h, then almost *L.L*-CFSE did not co-localized with LAMP1 at 6 h (**Figure 4B**). These results indicated that ROS decreased the degradation rate of antigen and promoted the release of antigen in GM-DCs through the phagosome-to-cytosol pathway.

To further investigate whether *L.L* enters GM-DCs through endocytosis, GM-DCs were treated with *L.L*-CFSE for 6 h in the presence or absence of cytochalasin D and analyzed by flow cytometry. As shown in **Supplemental Figure 5A**, cytochalasin D significantly reduced the amount of *L.L*-CFSE in GM-DCs in a dose-dependent manner. The subcellular localization of *L.L*-CFSE was detected in GM-DCs with or without 0.5 μg/ml cytochalasin D. After treatment for 6 h, cells were stained with EEA1, LAMP1 and DAPI and detected by confocal laser scanning microscopy. In the absence of cytochalasin D, lots of *L.L*-CFSE entered GM-DC and did not co-localize with EEA1 and LAMP1. This is consistent with the results in **Figure 4**. However, in the presence of cytochalasin D, *L.L*-CFSE did not appear in GM-GM-DC (**Supplemental Figure 5B**). Furthermore, mAb-TLR2 significantly reduced the amount of *L.L*-CFSE in GM-DCs (**Supplemental Figure 5C**). These results demonstrated that *L.L* entered GM-DC through endocytosis and released into cytoplasm through the phagosome-to-cytosol pathway.

### 3.5 OVA recombinant *L.L* enhances antigen cross-presentation by ROS production and proteasome

**Figure 5:**
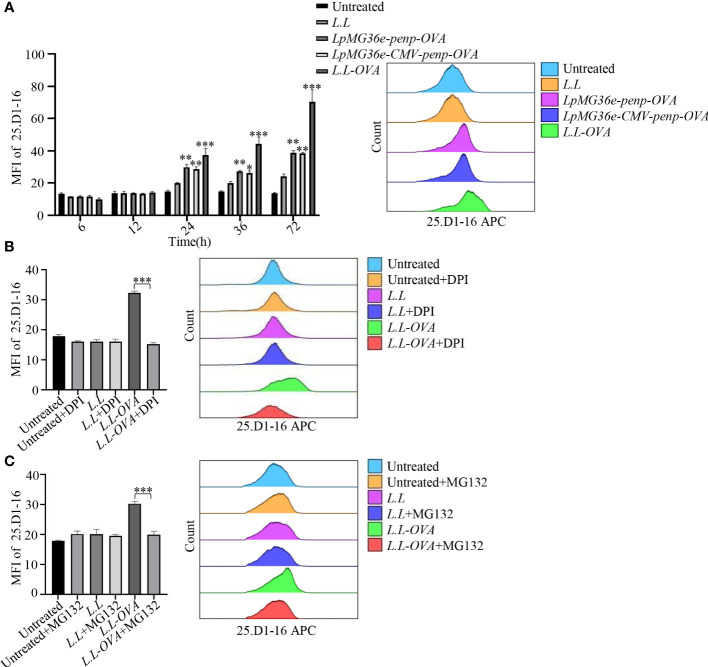
Antigen processing of *L.L-OVA* by GM-DCs. **(A)** The expression of MHC I-OVApep on GM-DCs was detected by flow cytometry after co-cultured with different OVA recombinant *L.L* for 6 h, 12 h, 24 h, 36 h and 72 h. **(B)** The effects of DPI (ROS inhibitor) on the expression of MHC I-OVApep in GM-DCs after co-cultured with *L.L-OVA* were detected by flow cytometry. GM-DCs were pretreated with DPI for 12 h, then co-treated with *L.L-OVA* for 24 h. **(C)** The effects of MG132 (proteasome inhibitor) on the expression of MHC I-OVApep in GM-DCs after co-cultured with *L.L-OVA* were detected by flow cytometry. GM-DCs were pretreated with 10 μg/ml MG132 for 30 min and co-treated with *L.L-OVA* for 24 h, then another 10 μg/ml MG132 was added after 12 h. * *p* < 0.05; ** *p* < 0.01; *** *p* < 0.001 compared to *L.L* group, or between indicated groups.

The GM-DC used in our experiment was cultured on day 7. However, we cannot exclude whether GM-DC or GM-CSF-derived macrophages are responsible for the observations with this type of culture. To further test whether OVA recombinant *L.L* enhances antigen cross-presentation, GM-DCs were treated with *LpMG36e-penp-OVA*, *LpMG36e-CMV-penp-OVA* and *L.L-OVA* for 6 h, 12 h, 24 h, 36 h and 72 h and stained with 25.D1-16-APC, which was used to specifically detect MHC I-OVApep complex on the surface of GM-DCs. As shown in **Figure 5A**, all the three OVA recombinant *L.L* significantly increased the fluorescence intensity of 25.D1-16 in a time-dependent manner and *L.L-OVA* generated the highest level of MHC I-OVApep complex. Therefore, *L.L-OVA* was selected for the next experiments. The roles of ROS production and proteasome in the generation of MHC I-OVApep complex were detected. GM-DCs were treated with *L.L-OVA* in the presence or absence of 5 μM DPI for 24 h and stained with 25.D1-16-APC. The fluorescence intensity of 25.D1-16 was significantly decreased by DPI (**Figure 5B**), indicating that ROS production induced by *L.L* promoted the release of OVA from phagosomes to cytoplasm. For the treatment of proteasome inhibitor, MG132, GM-DCs were pretreated with 10 μg/ml MG132 for 30 min and co-treated with *L.L-OVA* for 24 h, then another 10 μg/ml MG132 was added after 12 h. The fluorescence intensity of 25.D1-16 was significantly reduced by MG132 (**Figure 5C**), indicating that the released OVA was degraded by proteasome in cytoplasm. Moreover, the selected dose of DPI and MG132 did not induce the apoptosis and necrosis of GM-DCs after treatment for 24 h (**Supplemental Figure 6A**), and did not affect GM-DC migration *in vitro and vivo* (**Supplemental Figures 6B, C**). These results suggested that *L.L-OVA* enhanced antigen cross-presentation by ROS production and proteasome processing.

### 3.6 GM-DC vaccine prepared with *L.L-OVA* enhanced antigen-specific immune responses and antitumor efficacy

Our previous study showed that GM-DC vaccine prepared with peptides generated stronger cellular immune responses (31). However, MHC restriction of peptides limits their clinical use. GM-DCs have a poor endocytosis capacity for soluble protein. Therefore, we develop recombinant *L.L* expressing protein antigen as the new antigen delivery system for GM-DC vaccine. LPS is a TLR4 agonist to promote GM-DC maturation. LPS-treated GM-DCs pulsed with OVA protein or peptides were used as negative or positive controls to compare with *L.L-OVA*. The antigen-specific T cell responses were detected in mice immunized with these GM-DC vaccines. 14 days after boosting, inguinal LNs were isolated to analyze immune responses. Upon OVA protein stimulation, LPS+OVApep and *L.L-OVA* significantly increased the frequencies of OVA-specific CD4^+^IFN-γ^+^, CD8^+^IFN-γ^+^ and CD8^+^Granzyme B^+^ T cells compared with *L.L* group. *L.L-OVA*+MG132 and *L.L-OVA*+DPI significantly reduced the frequencies of CD4^+^IFN-γ^+^, CD8^+^IFN-γ^+^ and CD8^+^Granzyme B^+^ T cells compared with *L.L-OVA* group. This was consistent with the results in **Figure 5**. Importantly, *L.L-OVA* significantly enhanced OVA-specific CD8^+^IFN-γ^+^Granzyme B^+^ T cell responses compared with *L.L* group, but LPS+OVA and LPS+OVApep did not induce the responses (**Supplemental Figure 7**). These results indicated that GM-DC vaccine prepared with *L.L-OVA* enhanced antigen-specific immune responses.

**Figure 6:**
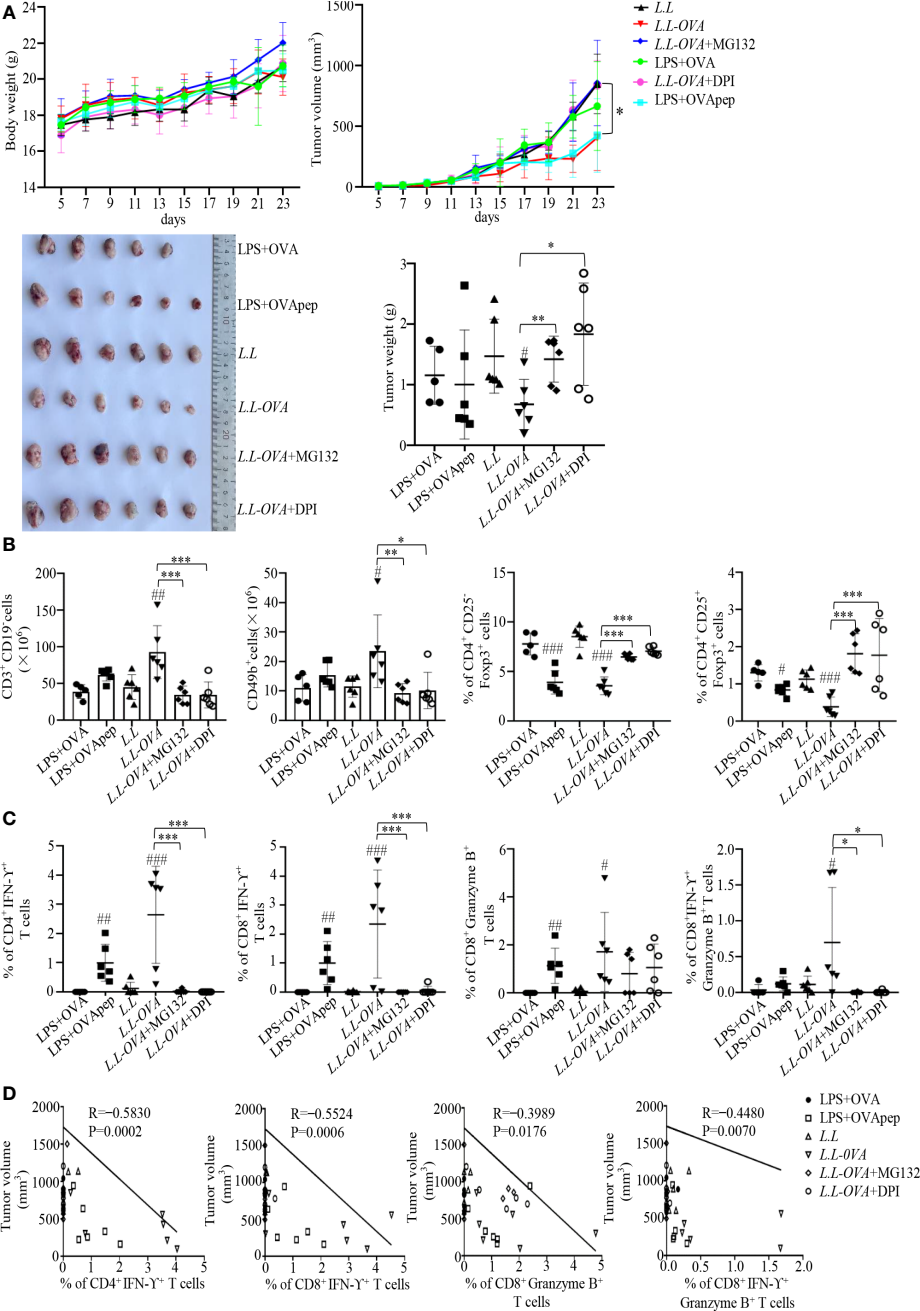
Antitumor effect *in vivo*. Mouse melanoma B16-OVA cells were subcutaneously injected into the right flank of C57BL/6 mice. **(A)** Mouse body weight, tumor volumes and tumor weight. **(B)** The frequencies of immune cells in spleens. CD3^+^CD19^-^ cells correspond to T cells; CD49b^+^ cells correspond to NK cells; CD4^+^CD25^-^Foxp3^+^ cells correspond to iTreg; CD4^+^CD25^+^Foxp3^+^ cells correspond to nTreg. **(C)** OVA-specific cellular responses in inguinal LNs. **(D)** The correlation of CD4^+^IFN-γ^+^, CD8^+^IFN-γ^+^, CD8^+^Granzyme B^+^ and CD8^+^IFN-γ^+^Granzyme B^+^ T cells with tumor volumes. # *p* < 0.05; ## *p* < 0.01; ### *p* < 0.001 compared to *L.L* group or LPS+OVA group, * *p* < 0.05; ** *p* < 0.01; *** *p* < 0.001 between indicated groups.

The antitumor effect of GM-DC vaccine prepared with *L.L-OVA* was further tested in B16-OVA tumor mouse model. All GM-DC vaccines did not affect the body weight of mice. Compared to *L.L* group, *L.L-OVA* and LPS+OVApep significantly inhibited tumor growth (**Supplemental Figure 8** and **Figure 6A**). *L.L-OVA* also significantly reduced tumor weight (**Figure 6A**). On day 24, spleens were isolated to detect immune responses. Compared with *L.L* group, *L.L-OVA* significantly increased the frequencies of T cells and NK cells in spleens of mice (**Figure 6B**). *L.L-OVA* and LPS+OVApep significantly decreased the frequencies of inducible regulatory T cells (iTregs: CD4^+^CD25^-^Foxp3^+^) and natural Tregs (nTregs: CD4^+^CD25^+^Foxp3^+^). However, the frequencies of iTregs and nTreg in *L.L-OVA*+MG132 and *L.L-OVA*+DPI groups were significantly increased compared with *L.L-OVA* group (**Figure 6B**). As expected, LPS+OVApep and *L.L-OVA* significantly enhanced OVA-specific CD4^+^IFN-γ^+^, CD8^+^IFN-γ^+^ and CD8^+^Granzyme B^+^ T cell responses compared with *L.L* group. *L.L-OVA* also significantly enhanced OVA-specific CD8^+^IFN-γ^+^Granzyme B^+^ T cell responses (**Figure 6C**). These immune responses are positively correlated with the inhibition of tumor growth (**Figure 6D**). *L.L-OVA*+MG132 and *L.L-OVA*+DPI significantly reduced CD4^+^IFN-γ^+^, CD8^+^IFN-γ^+^ and CD8^+^IFN-γ^+^Granzyme B^+^ T cell responses compared with *L.L-OVA* group (**Figure 6C**). These results indicated that GM-DC vaccine prepared with *L.L-OVA* had a strong antitumor effect.

## 4 Discussion

We found that *L.L* not only promoted GM-DC maturation, characterized by the upregulated expression of CD40, CD80, CD86, CCR7, MHC II, IL-12p40, TNF-α, IL-6 and IL-1β through TLR2 and its downstream MAPK and NF-κB signaling pathways, but also increased the production of ROS and NO in GM-DCs in a time and concentration dependent manner. It has been reported that ROS can affect the processing and degradation of antigen in phagocytosis (35). The high level of ROS inhibits the activity of proteolytic enzymes in phagocytosis (36) and mediates the rupture of phagosomes membrane, which not only reduces the degradation of antigen, but also promotes the exposure of antigen to the cytoplasm, prolongs the degradation time of antigen, and provides sufficient time for antigen cross-presentation of GM-DC (37). Consistently, we also observed that the addition of ROS scavengers (DPI and NAC) significantly reduced the amount of *L.L* in GM-DCs, suggesting the quickly degradation of antigen without ROS production. Moreover, *L.L* entered GM-DC through endocytosis and released into cytoplasm through the phagosome-to-cytosol pathway in a ROS dependent manner. In cytoplasm, proteasome can degrade proteins into peptides that can be presented by MHC I to enhance the antigen cross-presentation. The downregulated expression of MHC I-OVApep on GM-DCs further confirmed the result after *L.L-OVA* treatment in the presence of DPI and MG132. Importantly, DPI and MG132 significantly reduced the OVA-specific CD8^+^ T cell responses induced by *L.L-OVA* prepared GM-DC vaccine and abolished its antitumor effect in tumor mouse study. Therefore, *L.L* promoted GM-DC maturation and antigen cross-presentation through phagosome-to-cytosol pathway.

The types of immune responses induced by vaccines are closely related to the MHC gene haplotypes, which have a dramatic diversity in human population. Therefore, the peptide antigens have MHC restriction in the induction of immune responses for human being. Our previous studies have shown that GM-DC vaccine prepared with peptides can induce strong cellular immune responses and exhibits potential antitumor efficacy (38). To further carry forward the clinical use of GM-DC vaccine, *L.L* was used to deliver protein antigen for preparing GM-DC vaccine to avoid MHC restriction. In this study, different recombinant *L.L* expressed OVA were constructed, which were controlled by eukaryotic promoter CMV and/or prokaryotic promoter penp. We expected that *LpMG36e-CMV-penp-OVA* should produce higher level of MHC I-OVApep on GM-DCs due to the base expression under control of penp in *L.L* and the additional expression in GM-DC cytoplasm under control of CMV. However, we found that the level of MHC I-OVApep produced by *L.L-OVA* (pNZ8149*-penp-OVA*) treated GM-DCs was significantly higher than that produced by *LpMG36e-penp-OVA* and *LpMG36e-CMV-penp-OVA* treated GM-DCs. The possible reason might be that different vectors expressed different levels of OVA in *L.L*. In the future study, pNZ8149 vector will be used to construct recombinant *L.L* with eukaryotic OVA expression and prokaryotic and eukaryotic OVA expression to compare the immune responses.

GM-DC vaccine prepared with *L.L-OVA* not only induced strong OVA-specific cellular responses and increased the frequencies of T cells and NK cells but also decreased the frequencies of Tregs in spleen of mouse, which had a strong antitumor effect in the B16-OVA mouse tumor model. Unexpectedly, the addition of MG132 also reduced CD4^+^ T cell responses induced by GM-DC vaccine prepared with *L.L-OVA*. The possible reason might be that MG132 inhibited not only proteasome activity but also lysosomal protease activity to decrease the generation of MHC II-peptide complex, thus reducing the production of CD4^+^IFN-γ^+^ T cells. Consistently, we observed that the addition of MG132 significantly increased the fluorescence intensity of FITC-OVA encapsulated by gold nanoparticles in GM-DCs (**Supplemental Figure 9**). The addition of DPI rapidly inhibited the production of ROS in the phagosomes to promote the activation of proteolytic enzymes and rapid degradation of antigens, which might not be suitable for MHC II presentation, thus reducing the production of CD4^+^IFN-γ^+^ T cells.

GM-DC vaccine prepared with *L.L-OVA* induced strong antigen-specific immune responses and significantly inhibited B16-OVA tumor growth, which provided a new strategy for preparation of GM-DC vaccine. For other types of tumors, tumor associated antigens or neoantigens can be used to construct recombinant *L.L* to prepare GM-DC vaccine. For example, recombinant *L.L* expressing human papilloma virus E6 and E7 protein can be constructed to prepare GM-DC vaccine and treat cervical cancer, and GM-DC vaccine prepared with recombinant *L.L* expressing Her-2 can be used to treat breast cancer. Neoantigens can be identified by genomics and proteomics and used to construct recombinant *L.L* and prepare GM-DC vaccine.

Both *L.L* and LPS could induce high levels of IL-12 production in GM-DCs. Therefore, GM-DC vaccines prepared with *L.L* and LPS secreted high levels of IL-12 and induced Th1 and CTL responses. We did not determine whether *LL-OVA* and LPS-OVApep vaccines could influence IL-12 production by cDC1s in the LNs because *L.L* and LPS were washed out. Although we speculate these GM-DC vaccines may not affect other GM-DCs *in vivo*, it’s worth to determine it in future experiments. In addition, a well-known impact of cancer is a reduction in anticancer cDC1s and in increase in myeloid regulatory DCs with high PDL-1 and PDL-2 expression. However, we do not know whether *LL-OVA* impacts cDC1s, cDC2s and regulatory DCs in the LNs. We will systemically analyze it in future experiments.

## 5 Conclusion

*L.L* promoted GM-DC maturation through TLR2 and its downstream MAPK and NF-κB signaling pathways, and induced high levels of ROS to promote GM-DC phagosomes membrane rupture, antigen release into the cytoplasm, and antigen cross-presentation through the phagosome-to-cytosol pathway. GM-DC vaccine prepared with *L.L-OVA* induced strong antigen-specific immune responses and inhibited B16-OVA tumor growth. This study demonstrated that *L.L* is a potential antigen deliver candidate that promotes GM-DC maturation and antigen cross-presentation.

## Data availability statement

The original contributions presented in the study are included in the article/**Supplementary Material**. Further inquiries can be directed to the corresponding author.

## Ethics statement

The animal study was reviewed and approved by Ethics Committee of Xinjiang University, Xinjiang University (XJUAE-2019-027).

## Author contributions

JL designed and revised the manuscript. TZ carried out experiments and wrote the manuscript. XW, SH, YW and SC participated in data analysis. YL and AA participated in experiment design and help with materials. All authors read and approved the final manuscript.
