# Supplementary material for: Dendritic cell-based vaccine prepared with recombinant Lactococcus lactis enhances antigen cross-presentation and antitumor efficacy through ROS production
Source: Front Immunol. 2023 Aug 30;14:1208349. doi: 10.3389/fimmu.2023.1208349 (PMC10498461; doi:10.3389/fimmu.2023.1208349)
Supplement: Supplementary file 1 [file DataSheet_1.docx]

Supplementary Material

**Dendritic cell-based vaccine prepared with** **recombinant** ***Lactococcus lactis* enhances antigen cross-presentation and antitumor efficacy through ROS production**

**Tingting Zhang, Xianxian Wei, Yijie Li, Shuai Huang, Yulin Wu, Shanshan Cai, Adila Aipire, Jinyao Li***

*** Corresponding author**: Jinyao Li (e-mail: [ljyxju@xju.edu.cn](mailto:ljyxju@xju.edu.cn)).

**
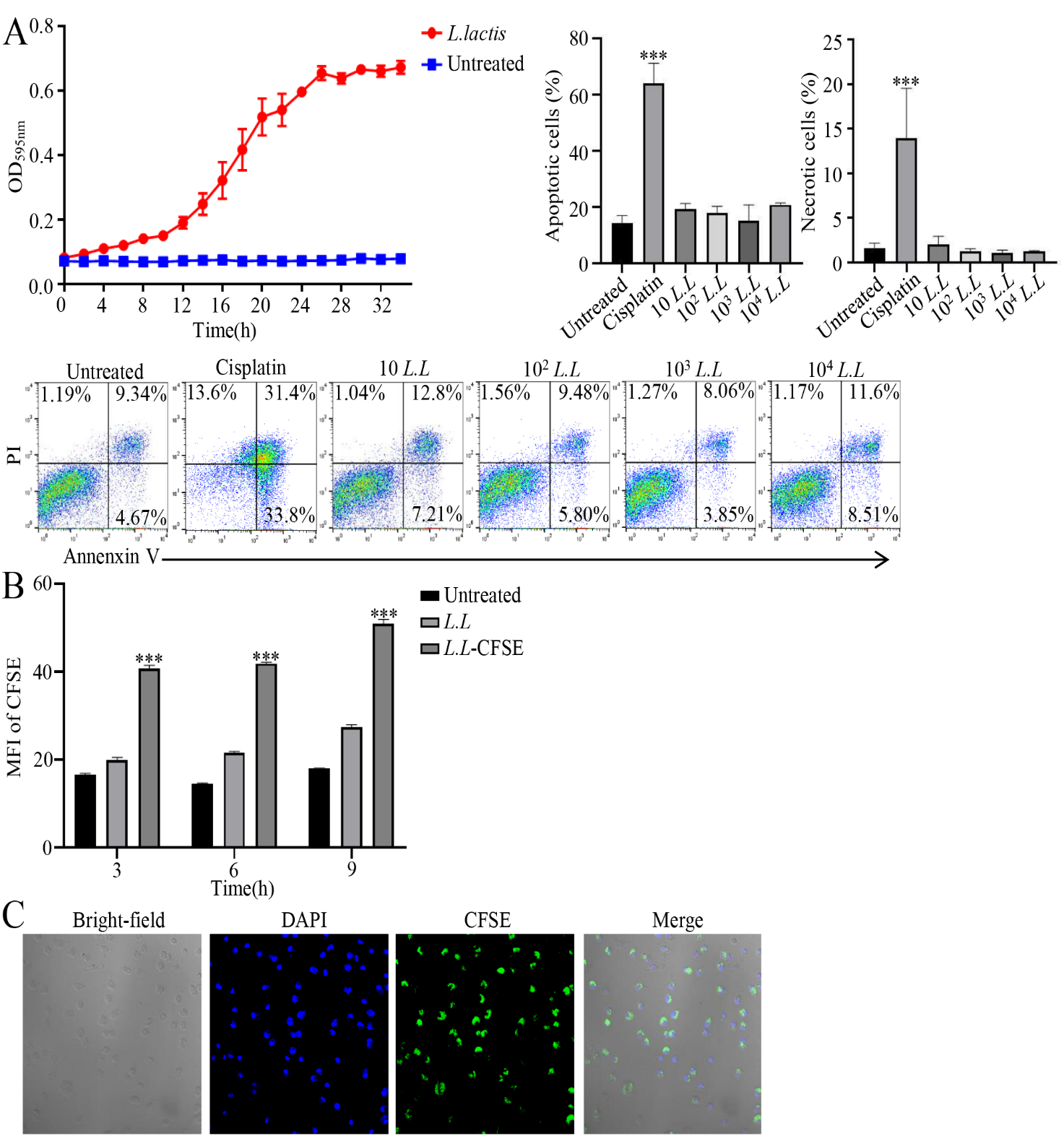
**

**Supplemental Fig. 1.** **The effect of *L.L* on GM-DC viability and phagocytosis of *L.L* by GM-DC.** (A) Determination of growth curve of *L.L* and effect of *L.L* on GM-DC viability. (B) Phagocytosis of *L.L* by GM-DC was analyzed with flow cytometry. (C) The subcellular localization of *L.L*-CFSE in GM-DCs was detected by confocal laser scanning microscopy. *** *p* < 0.001 compared to untreated group or control group.


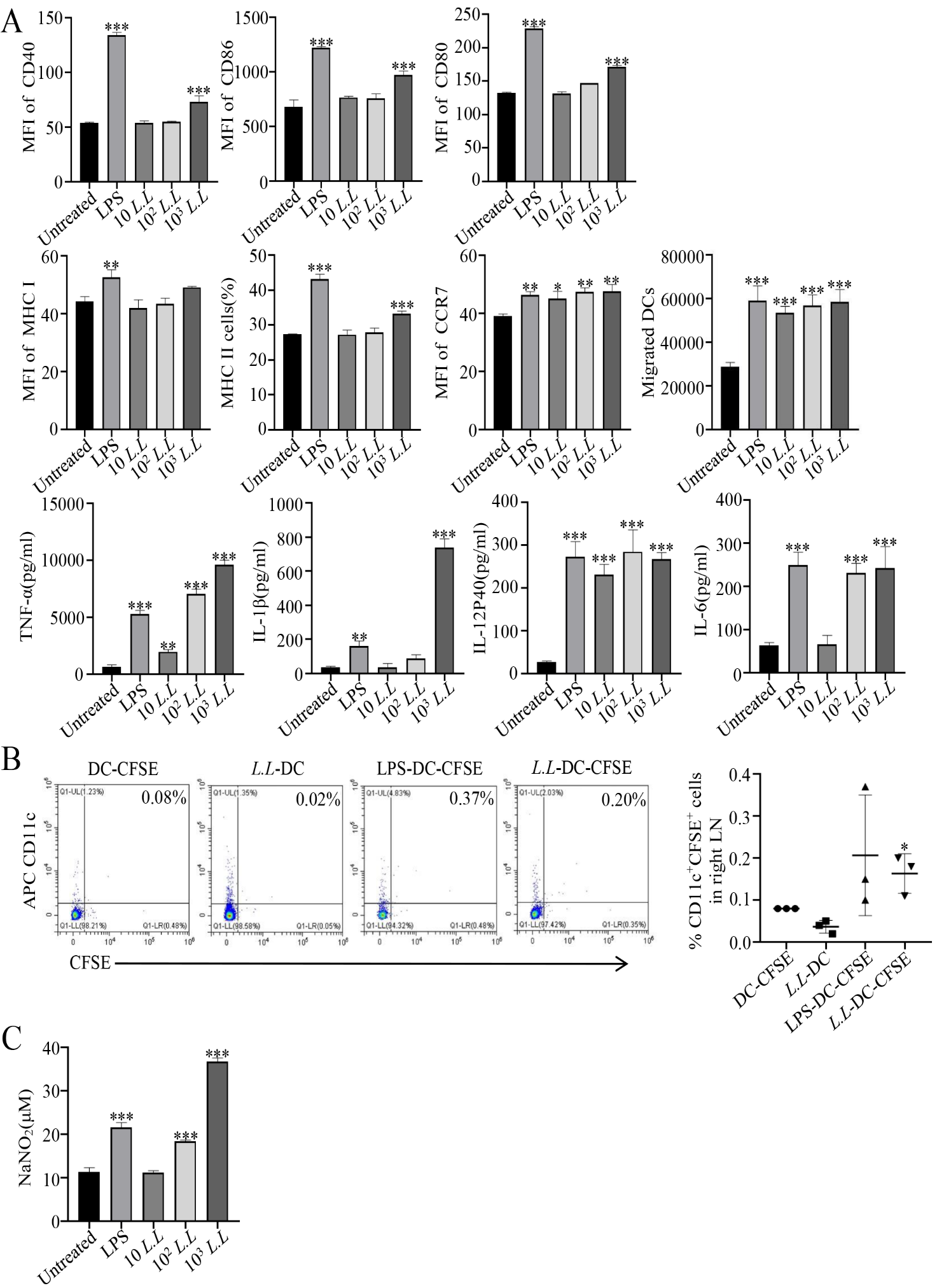


**Supplemental Fig. 2. GM-DC activation induced by *L.L*.** (A) The expression of surface molecules and cytokines in GM-DCs was detected by flow cytometry and ELISA, respectively. The MFI (mean fluorescence intensity) of surface molecules was shown. GM-DC migration *in vitro* was detected by transwell. (B) GM-DC migration *in vivo*. The frequencies of CFSE^+^ GM-DCs in draining LN were shown. (C) NO production of GM-DC was measured by Griess. * *p* < 0.05; ** *p* < 0.01; *** *p* < 0.001 compared to untreated group or control group.


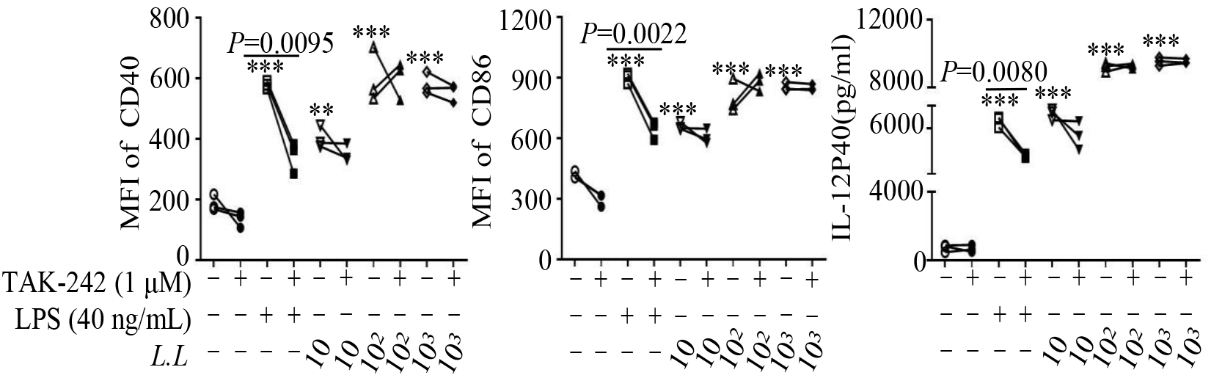


**Supplemental Fig. 3. The effect of TLR4 inhibitor TAK-242 on GM-DC maturation treated with *L.L*. GM-**DCs were pretreated with TAK-242 for 1 h, and then treated with LPS and *L.L* for 24 h. The expression of CD40 and CD86 was tested by flow cytometry. The IL-12p40 secretion was measured by ELISA. ** *p* < 0.01; *** *p* < 0.001 compared to untreated group.


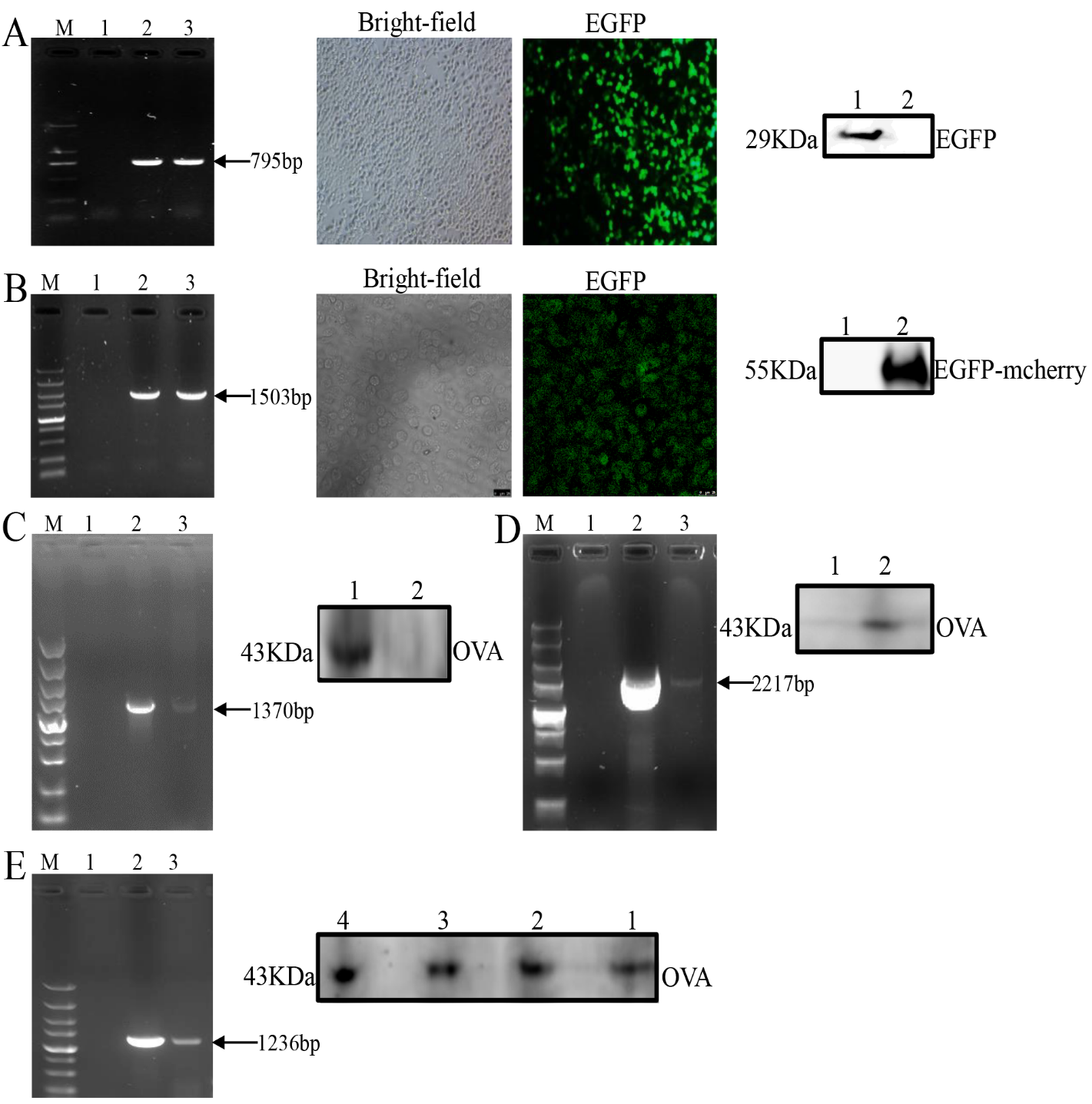


**Supplemental Fig. 4. Construction and identification of recombinant vectors.** (A) EGFP protein expression of pMG36e-*RFP/eGFP* (358) and identification of *L358*. The expression of EGFP protein in 293T cells transfected with pMG36e-*RFP/eGFP* (358) was detected by Western blot and confocal laser scanning microscopy, respectively. For Western blot, 1: protein from 293T cells transfected with pMG36e-*RFP/eGFP* (358), 2: protein from 293T cells. *L358* PCR identification. 1: DNA from *L.L*, 2: pMG36e-*RFP/eGFP* (358) plasmid, 3: DNA from *L358*, M: 2000bp DNA marker. (B) The identification of *LEGFP-mcherry* and its protein expression. *LEGFP-mcherry* PCR identification. 1: DNA from *L.L*, 2: pMG36e-*EGFP-mcherry* plasmid, 3: DNA from *LEGFP-mcherry*, M: 5000bp DNA marker. The protein expression of *LEGFP-mcherry* was detected by Western blot. 1: protein from *L.L*, 2: protein from *LEGFP-mcherry*. The fluorescence of EGFP was detected by confocal laser scanning microscopy after co-culture of *LEGFP-mcherry* and GM-DCs for 6 h. (C) The identification of *LpMG36e-penp-OVA* and its protein expression. *LpMG36e-penp-OVA* PCR identification. 1: DNA from *L.L*, 2: pMG36e-*penp-OVA* plasmid, 3: DNA from *LpMG36e-penp-OVA*, M: 5000bp DNA marker. The protein expression of *LpMG36e-penp-OVA* was detected by Western blot. 1: protein from *LpMG36e-penp-OVA*, 2: protein from *L.L*. (D) OVA protein expression of pMG36e-*CMV-penp-OVA* and *LpMG36e-CMV-penp-OVA* identification. The protein expression of pMG36e-CMV-penp-OVA was detected by Western blot. 1: protein from 293T cells, 2: protein from 293T cells transfected with pMG36e-*CMV-penp-OVA*. *LpMG36e-CMV-penp-OVA* PCR identification. 1: DNA from *L.L*, 2: pMG36e-*CMV-penp-OVA* plasmid, 3: DNA from *LpMG36e-CMV-penp-OVA*. (E) The identification of *L.L-OVA* and its protein expression. *L.L-OVA* PCR identification. 1: DNA from *L.L*, 2: pNZ8149-*penp-OVA* plasmid, 3: DNA from *L.L-OVA*, M: 5000bp DNA marker. The protein expression of *L.L-OVA* was detected by Western blot. 1: protein from *L.L-OVA* without induction, 2-4: protein from *L.L-OVA* after induction for 9 h, 12 h and 24 h, respectively.

**
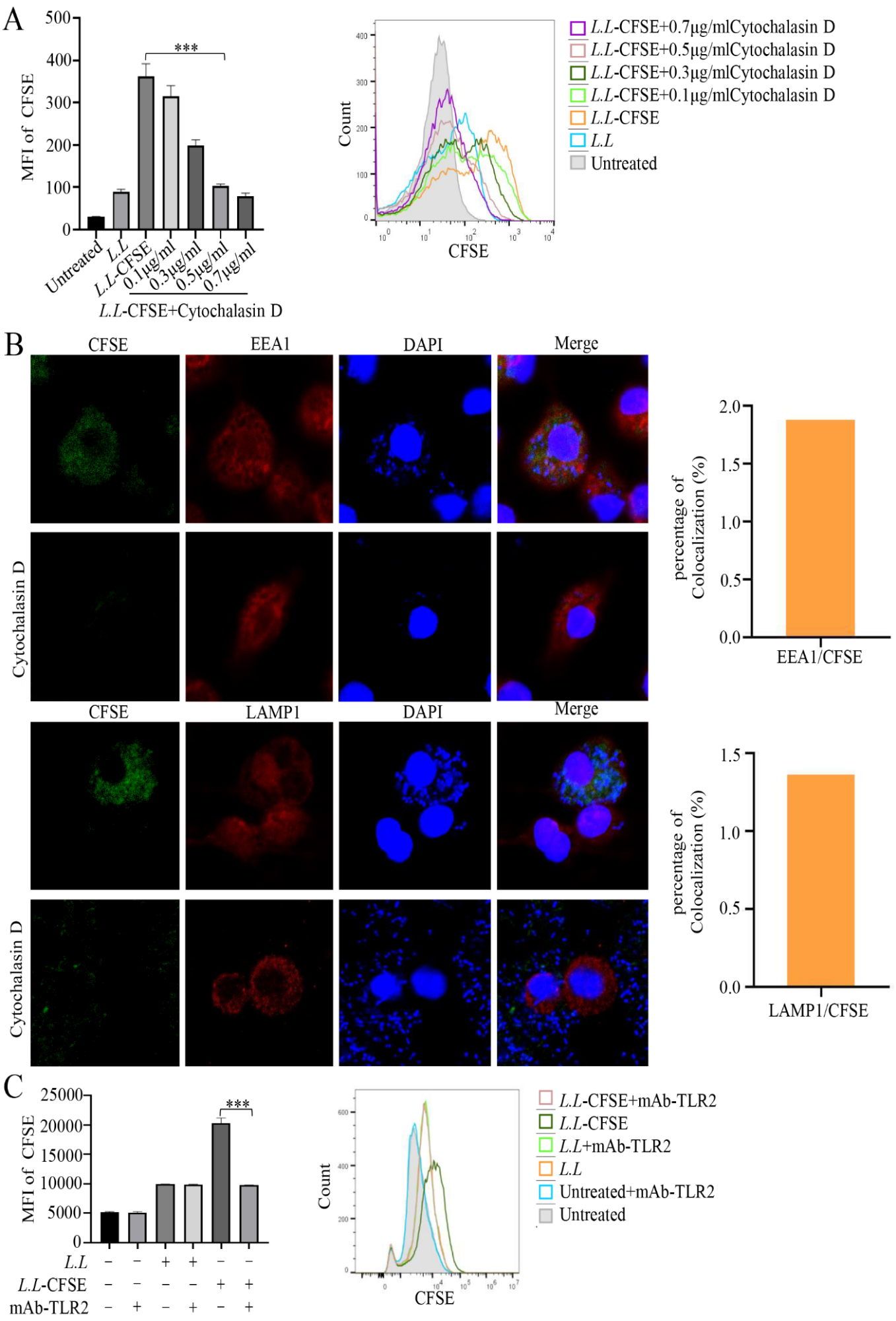
**

**Supplemental Fig. 5. *L.L* entered GM-DCs through endocytosis.** (A) The effect of different concentrations of cytochalasin D (endocytosis inhibitor) on GM-DC phagocytosis of *L.L*-CFSE was detected by flow cytometry. GM-DCs were pretreated with different doses of cytochalasin D for 12 h, and then treated with *L.L*-CFSE for 24 h. (B) The co-localization of *L.L*-CFSE with EEA1 and LAMP1 was observed by confocal laser scanning microscopy with or without 0.5 μg/ml cytochalasin D pretreatment. The quantification of colocalization was shown in right panel. (C) The effect of mAb-TLR2 on GM-DC phagocytosis of *L.L*-CFSE was detected by flow cytometry. GM-DCs were pretreated with mAb-TLR2 (blocking antibody) for 1 h, and then treated with *L.L*-CFSE for 24 h. *** *p* < 0.001 compared to indicated groups.


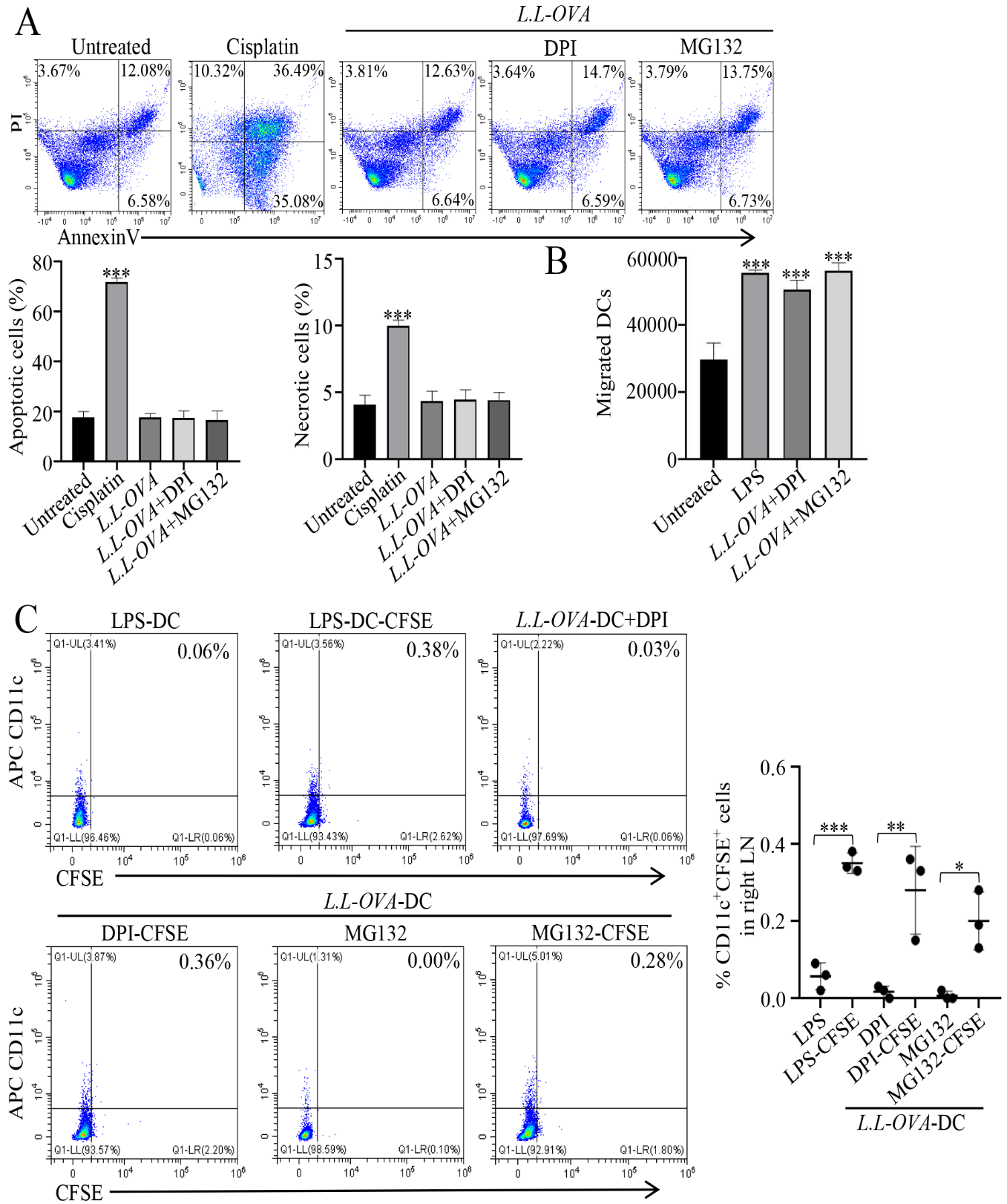


**Supplemental Fig. 6. The effects of DPI (ROS inhibitor) and MG132 (proteasome inhibitor) on GM-DC viability and migration.** GM-DCs were pretreated with DPI for 12 h or MG132 for 30 min, followed by another 10 μg/ml MG132 after 12 h, and then treated with *LL-OVA* for 24 h. (A) GM-DC viability was analyzed by flow cytometry after AnnexinV-FITC/PI staining. (B) GM-DC migration *in vitro* was detected by transwell. (C) GM-DC migration *in vivo* was detected by flow cytometry. * *p* < 0.05; ** *p* < 0.01; *** *p* < 0.001 compared to untreated group, or between indicated groups.


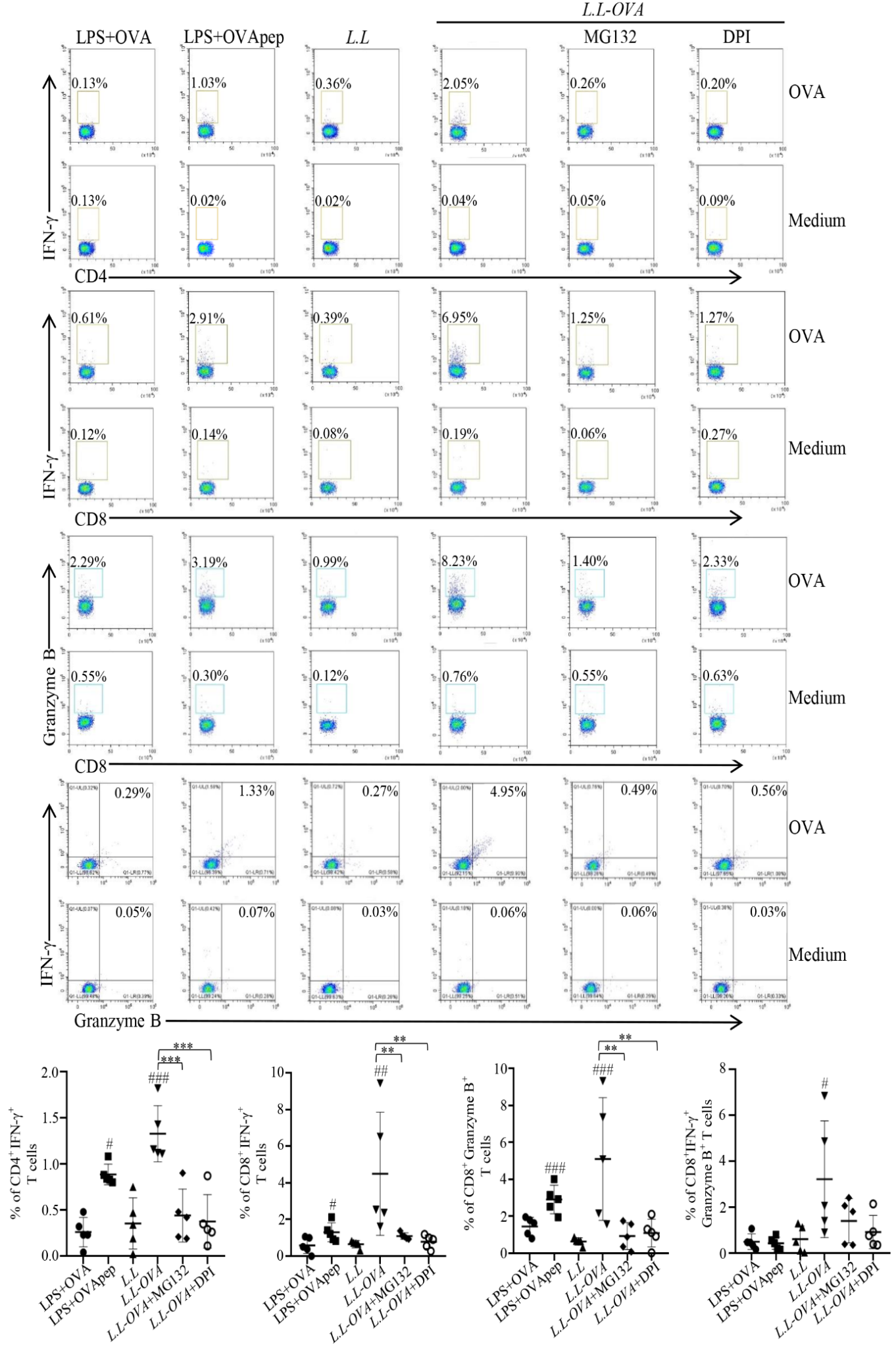


**Supplemental Fig. 7. The immune responses induced by GM-DC vaccine prepared with *L.L-OVA*.** After immunization, [inguinal LNs](javascript:;) were isolated to detect OVA-specific cellular immune responses. The frequencies of CD4^+^IFN-γ^+^, CD8^+^IFN-γ^+^, CD8^+^Granzyme B^+^ and CD8^+^IFN-γ^+^Granzyme B^+^ T cells were shown. # *p* < 0.05; ## *p* < 0.01; ### *p* < 0.001 compared to *L.L* group, * *p* < 0.05; ** *p* < 0.01; *** *p* < 0.001 between indicated groups.


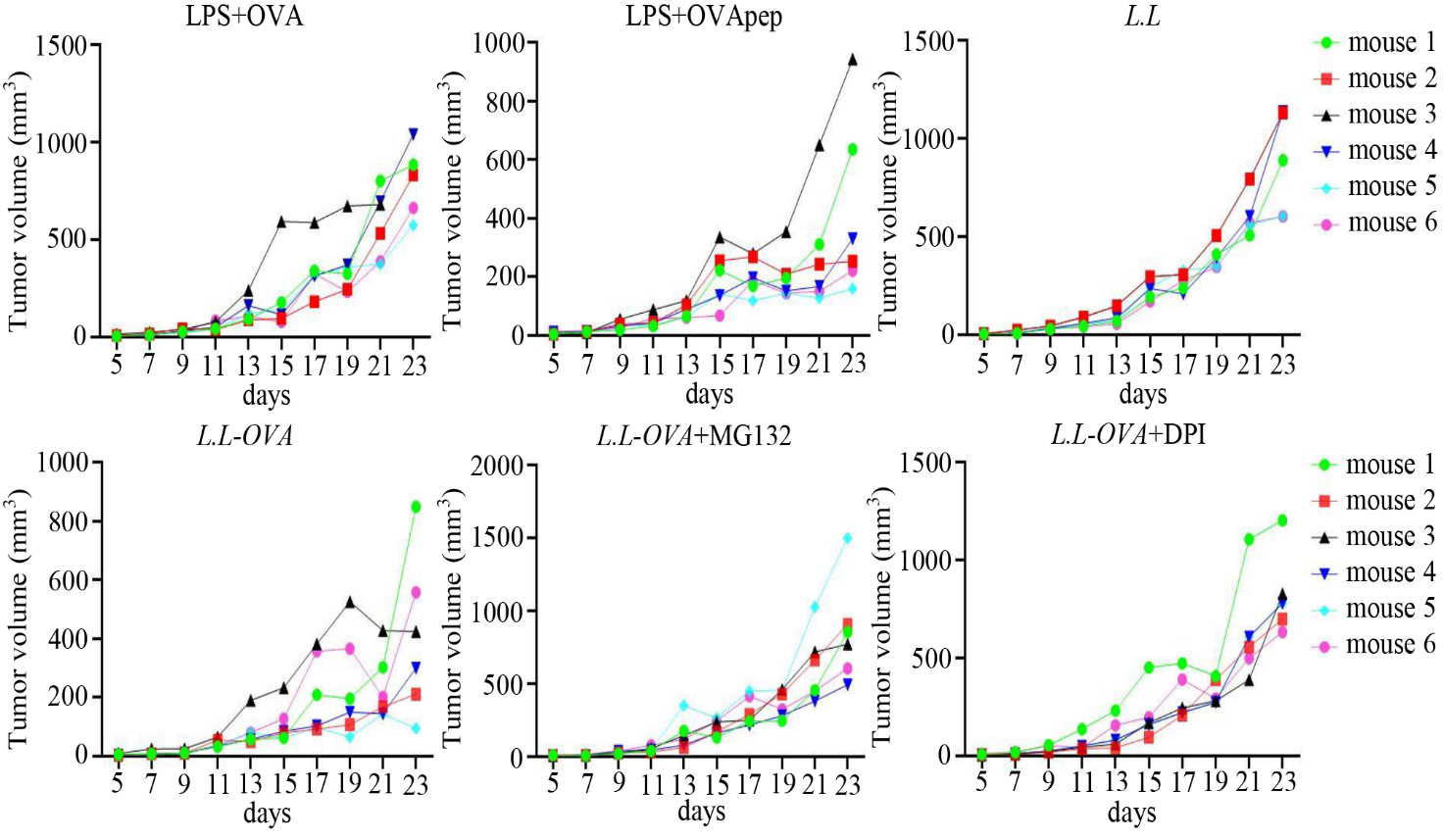


**Supplemental Fig. 8.** The separate tumor volumes growth curve corresponds to Fig. 6A.


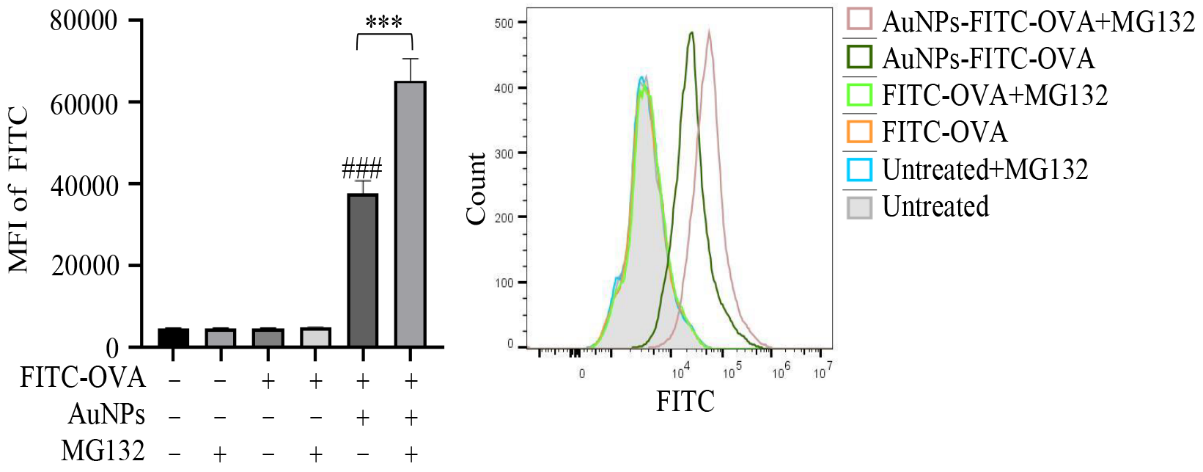


**Supplemental Fig. 9.** **The effect of MG132 (proteasome inhibitor) on the fluorescence intensity of FITC-OVA coated by AuNPs in GM-DCs.** GM-DCs were pretreated with 10 μg/ml MG132 for 30 min and co-treated with 100 μg/ml FITC-OVA for 24 h, then another 10 μg/ml MG132 was added after 12 h. ### *p* < 0.001 compared to untreated group, *** *p* < 0.001 between indicated groups.
